# Basal Cisternostomy for Severe TBI: Surgical Technique and Cadaveric Dissection

**DOI:** 10.3389/fsurg.2022.915818

**Published:** 2022-05-06

**Authors:** L. Giammattei, D. Starnoni, M. Messerer, R. T. Daniel

**Affiliations:** ^1^ Department of Neurosurgery, Lausanne University Hospital, Lausanne, Switzerland; ^2^ Faculty of Biology and Medicine, University of Lausanne, Lausanne, Switzerland

**Keywords:** severe brain trauma, decompressive craniectomy (DC), skull base, cisternostomy, cadaveric dissection

## Abstract

**Introduction:**

Cisternostomy is emerging as a novel surgical technique in the setting of severe brain trauma. Different surgical techniques have been proposed with a variable degree of epidural bone work. We present here the surgical technique as it is currently performed in our Institution.

**Methods:**

Anatomical dissection of one adult cadaveric head, injected and non-formalin fixed was perfomed. A large right fronto-temporo-parietal craniotomy was accomplished. Extradural sphenoidal drilling till opening of the superior orbital fissure was performed. The microsurgical anatomy of basal cisternostomy was then explored.

**Results:**

A step by step description of the surgical technique, enriched with cadaveric and intraoperative images, was made.

**Conclusion:**

Basal cisternostomy is a promising surgical technique that does not necessarily include complex surgical maneuvers. Trained neurosurgeon can safely implement it in their clinical practice.

## Introduction

Severe traumatic brain injury (TBI) is a life-threatening condition which is associated with substantial morbidity and mortality (1). The pathogenesis of TBI includes a primary injury related to a physical injury to the brain and a delayed secondary injury caused by the subsequent molecular, chemical and inflammatory cascades that can result in brain oedema, ischemia and intracranial hypertension. Cisternostomy is a novel surgical technique that has been proposed to prevent the development of secondary brain injury and treat associated increase in intracranial pressure (2, 3). A previous clinical study of our group (4) has showed that adjuvant cisternostomy is associated with an improved outcome (both at early and long term), improved brain oxygenation, better control of ICP and shorter ICU stay when compared to standard decompressive craniectomy (DC). A recent randomized trial by Chandra et al. (5) has also confirmed the benefit of cisternostomy in terms of outcome and ICP control when compared to standard DC. However, discordances exist between the different authors concerning the surgical technique (6). The aim of this study is to illustrate in details the surgical technique employed in our Institution with the support of cadaveric dissection and intraoperative surgical images in order to guide neurosurgeons interested in implementing this promising technique in their clinical practice.

## Methods

### Surgical Case Series

The following described surgical technique has been employed in a standardized manner in a previously published retrospective surgical series of 18 patients treated with adjuvant cisternostomy (AC group) that were compared to 22 patients that underwent decompressive craniectomy (DC group) in case of severe brain trauma (4). The AC group included 13 patients that were surgically treated as primary procedure (patients presenting a unilateral mass effect lesion) and 5 patients that were treated as secondary procedure (refractory intracranial hypertension despite medical therapy). The DC group included 11 patients that treated as primary procedure and 11 patients treated as secondary procedure. The surgical procedure of the AC group included cisternostomy as an adjunct procedure to standard DC. AC was associated with significant shorter duration of mechanical ventilation and ICU stay, as well as better Glasgow coma scale at discharge. The outcome difference was particularly relevant when AC was performed as primary procedure. Patients in the AC group also had significant lower average post-surgical ICP values, higher PbO2 values and required less osmotic treatments as compared with those treated with DC alone.

### Cadaveric Dissections

The anatomical dissections were performed at the neuroanatomy laboratory of the Lausanne University Hospital, Switzerland. The surgical anatomy of cisternostomy was described using 1 adult cadaveric head injected with colored latex, non formalin-fixed. The procedure was performed with standard microsurgical instruments, high-speed drill (Midas Rex; Medtronic, Minneapolis, MN, USA) and a surgical microscope (Leica Camera AG, Wetzler, Germany). The operative video was recorded using a 2D/4K camera (Karl Storz GmbH, KG, Tuttlingen, Germany) connected to the microscope. The head was fixed with a three-pins Mayfield clamp, contra laterally turned to 60 for the soft tissues dissection and craniotomy. The contralateral turn was then changed to 30 in order to perform the sphenoidal drilling and the cisternostomy. The microscope was used to perform the sphenoidal drilling, to open the basal cisterns and to place the cisternal drain. Each step of the surgical technique is described in details. Tips and tricks of each step according to the experience of our group are also presented, with accompanying cadaveric dissections and intraoperative illustrative pictures. A video illustrating in details the cadaveric dissection is also added (video 1).

## Surgical Technique

### Positioning

The patient is placed in the supine position with 30 degrees of head elevation to decrease the intracranial pressure. The head, fixed in a skull-clap system, is contralaterally turned of 60 degree and extended in order to have the malar eminence as highest point of the specimen. During surgery the absence of jugular stenosis on both sides should be verified. This contralateral turn will facilitate the first part of surgery that approximates that of a standard trauma-flap procedure. It is important to verify that the patient is adequately secured onto the table to enable table tilt to obtain 30 degree of head turn in order to facilitate the sphenoidal drilling and the cisternal opening.

### Skin Incision and Soft Tissue dissection

**Figure 1:**
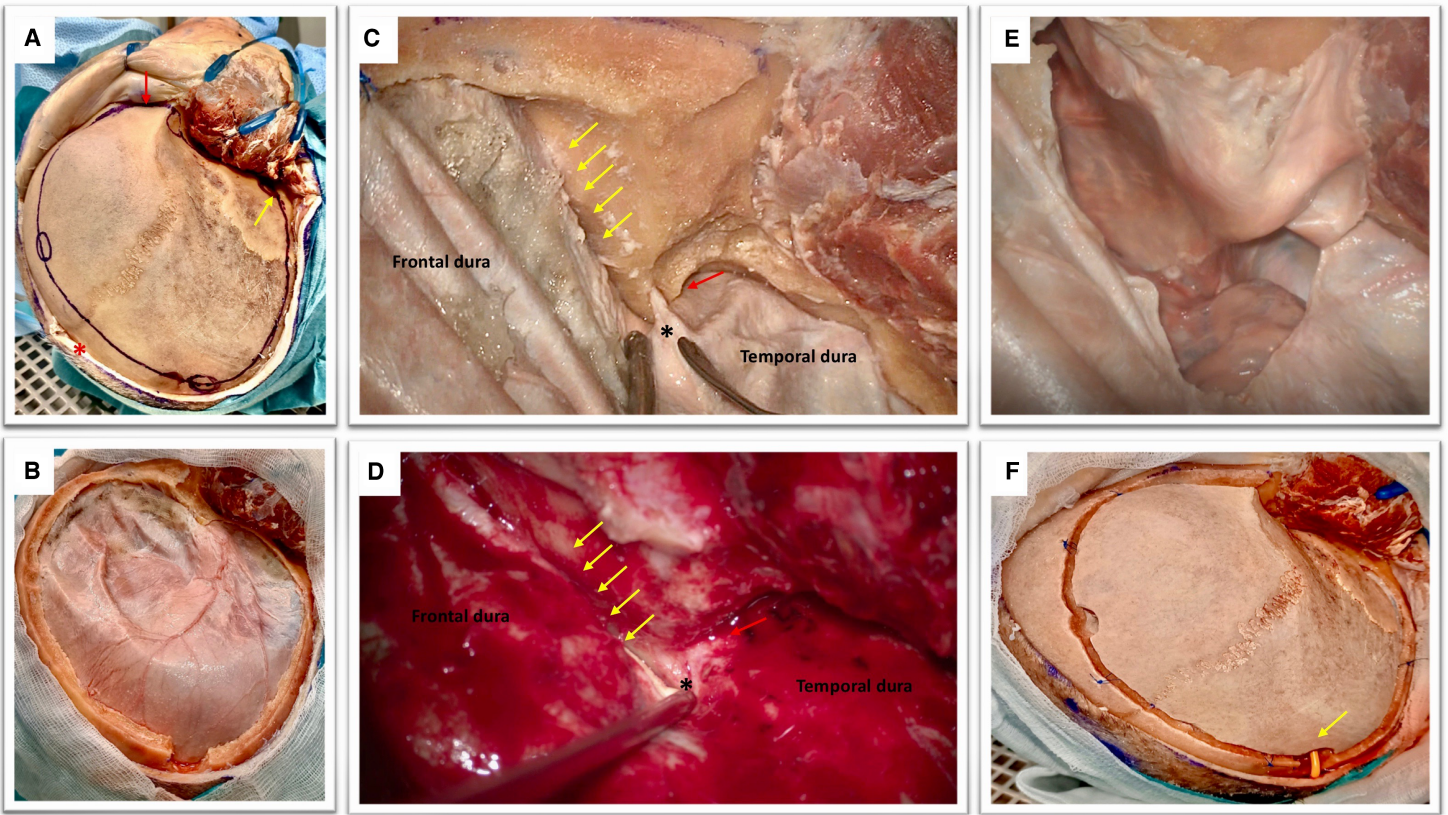
(**A**) Cadaveric dissection obtained after the elevation of soft tissues. The temporal muscle is retracted anteriorly with multiple hooks. The coronal suture is seen as well as the fontal, parietal, the squamous part of the temporal bones, the key hole and the orbital rim (red arrow). Please note the position of the midline (red asterisk) and the root of the zygoma (yellow arrow) that can be easily palpated in order to perform a craniotomy that is flush with the middle fossa. Note that the soft tissues are dissected till obtaining a visualization of the supra-orbital nerve and the orbital rim (red arrow).This is an essential step to perform a basal craniotomy that will enable the subsequent intradural elevation of the frontal lobe. Please note the position of the frontal and parietal burr holes that should be at a proper distance from the midline (approximately 2 cm) to avoid the risk of injury to the superior sagittal sinus. The key hole has an antero-superior location with respect to the pterion and should avoid transgression of the orbit with an appropriate direction of the perforator that should point in a posterior direction. (**B**) The obtained craniotomy allows an adequate fronto-basal access that would be increased by the epidural sphenoid drilling. The remaining part of the greater wing of the temporal bone is also drilled in order to expose the middle fossa floor and rapidly provide temporal lobe decompression. (**C**,**D**) Epidural sphenoidal drilling is accomplished by flattening the skull base to obtain a lateral subfrontal access (according to the direction of the yellow arrows) and continued till the opening of the superior orbital fissure (red arrow). The meningo-orbital band is identified (black asterisk); this is a dural band that tethers the frontotemporal dura to the orbit and is attached to the lateral part of the superior orbital fissure. The epidural drilling is usually stopped at this point. (**E**) A basal durotomy is performed and this small dural flap is antero-laterally retracted to increase the skull base view. (**F**) If the bone flap is replaced, care should be taken to ensure that the subcutaneously tunneled cisternal drain is not kinked (yellow arrow).

A frontotemporal skin incision is performed in order to give access to frontal, parietal bone and temporal bones including the anterolateral skull base. The incision often goes beyond the midline to obtain a basal craniotomy. The inferior limit of this skin incision should be at the level of the zygoma and vey next to the tragus in order to avoid injury to the branches of the facial nerve. A monolayer myocutaneous flap is elevated through subperiosteal dissection. The muscle bulk is reflected anterolaterally and kept retracted with multiple small hooks. The dissection should proceed till identification of the orbital rim, supraorbital nerve and possibly the root of the zygoma inferiorly that will give a precise idea of the position of the middle fossa floor (Figure 1A).

### Craniotomy

Multiple burr holes are performed: one at the level of the key-hole, one anterior and one posterior to the coronal suture, paying attention to be at least 2 cm far away from the midline, one at the level of the parietal bone, one at the level of the squamous part of the temporal bone just superior to the root of the zygoma. A large craniotomy (15 × 12 cm in diameters) is obtained with the aid of a craniotome (Figure 1B). The craniotomy should be basal and the surgeon must pay attention to avoid entering the orbit. The supraorbital nerve can be considered as a good anatomical landmark to help surgeon to design a craniotomy that would allow a lateral subfrontal access. The bone flap is elevated and epidural bleeding is controlled using multiple dural tenting sutures.

### Epidural Drilling

The surgical table is then modified in order to have the head turned of 30 degree to facilitate anatomical orientation for the sphenoidal drilling and the intradural phase. Considering the clinical contest of raised intracranial hypertension it is important to focus first on the last part of remaining squamous part of the temporal bone and the greater sphenoid wing. It is very important to obtain a craniotomy that is flush with the middle fossa. This will enable to relieve the pressure on the temporal lobe. The temporal dura and frontal dura are then gently dissected from the skull base avoiding unintentional pressure onto the brain. The second step of this epidural drilling is now to shave the orbital roof in order to enable the surgeon to obtain a trajectory that is parallel to the skull base thus reducing frontal lobe manipulation. The third steps consists of drilling the sphenoidal bone till exposure of the meningo-orbital band and opening of the SOF (Figure 1C,D).

### Dural Opening

A small semicircular opening of the dura is perfomed staying close to the basal part of the craniotomy (Figure 1E). Dural tack-up sutures are applied in order to avoid an epidural bleeding that can perturb the intradural phase. The dura over the convexity can be opened in a later stage if needed (i.e., evacuation of an hematoma, removal of cerebral contusions) after the accomplishment of cisternostomy.

### Opening of the Basal Cisterns and Lamina Terminalis

**Figure 2:**
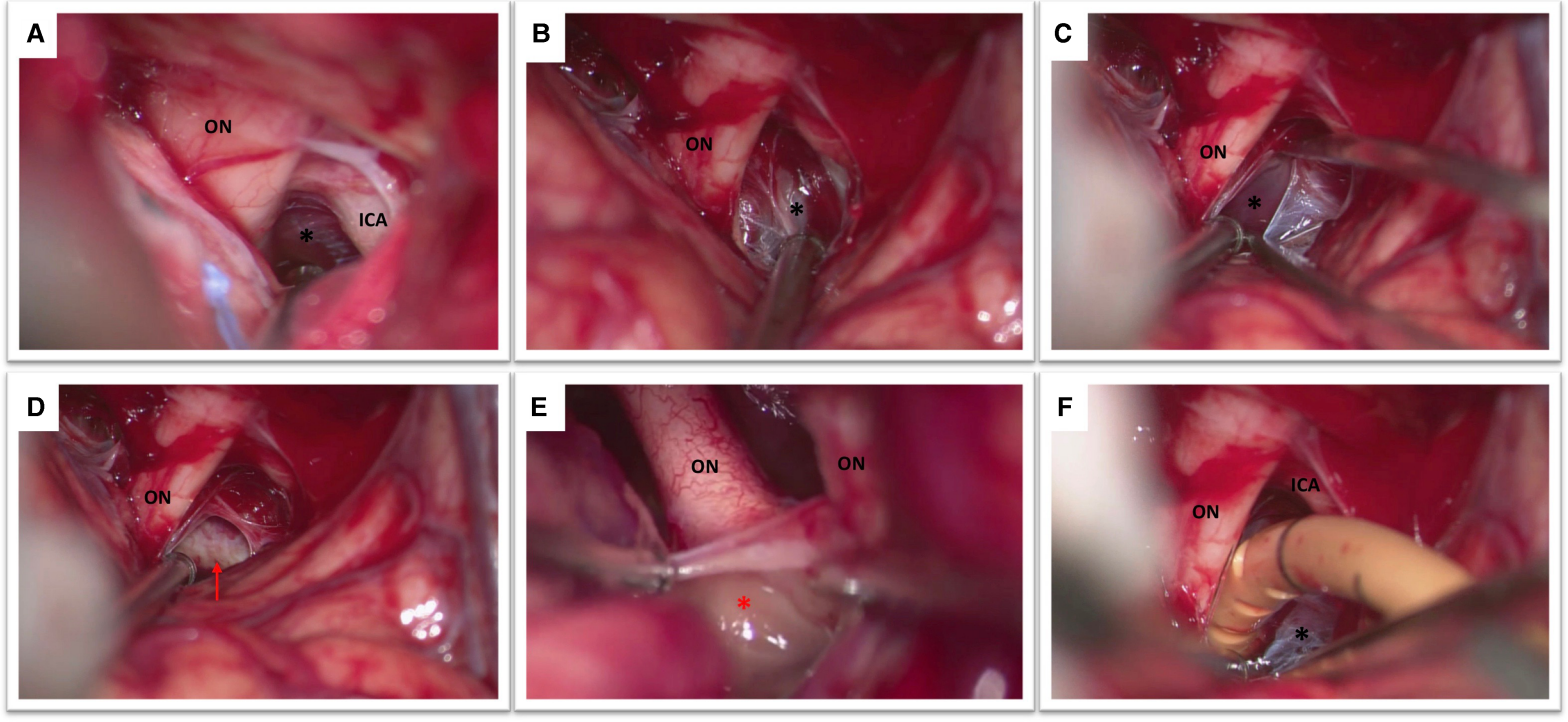
(**A**) This figure shows the view obtained after the opening of the optico-carotid cistern with optic nerve (ON), internal carotid artery (ICA) and Liliequist’s membrane (black asterisk). (**B**) The optic nerve (ON) and the diencephalic leaf of the Liliequist’s membrane (black asterisk) are seen. (**C**) The diencephalic leaf is opened enabling the exposure of the mesencephalic leaf (black asterisk). (**D**) The dorsum sellae (red arrow) becomes clearly visible (**E**) Both optic nerves and the lamina terminalis (red asterisk) are visible in this picture. (**F**) Note the position of the catheter which is placed in between the optic nerve (ON) and the internal carotid artery (ICA) passing through Liliequist’s membrane (black asterisk) into the prepontine cistern.

The first step is represented by obtaining a correct orientation of the operative microscope that allows a surgical view parallel to the skull base. To achieve a lateral subfrontal approach, a certain amount of frontal lobe manipulation is required. As a general rule the light of the operative microscope should be directed half on the brain and half on the skull base. The frontal lobe elevation should be performed very gently, cottonoids can be employed to protect the brain and to dry the surgical field in case of profuse intraoperative venous bleeding. It is also important to be careful with the distal part of the surgical intruments that can produce also iatrogenic contusions of the frontal lobe. Eventual component of hematoma that is on the way to the cisterns can be aspirated to reduce the intracranial pressure and facilitate the procedure. The olfactory nerve will inevitably cross the optic nerve; this is a very useful landmark to precisely localize the optic nerve and the internal carotid artery. The first cistern to be addressed is the optico-carotid cistern. Once the optico-carotid cistern has been opened the microscope should be oriented in a more antero-posterior direction in order to expose and open the Liliequist’s membrane (2A). The Liliequist’s membrane is usually composed by two leaves: the diencephalic and the mesencephalic one (Figure 2B–E). The homolateral optic nerve should be followed posteriorly onto the chiasm and optic tract to identify and open the lamina terminalis (Figure 2E). A gentle mobilization of the homolateral A1 segment is in general required to perform this last task.

### Cisternal Drain Positioning

A standard EVD drain is first subcutaneously tunneled and then the drain is inserted under the microscope at the level of the optico-carotid cistern possibly reaching the prepontine cistern. The position of the drain (the distal end) is verified under the microscope (Figure 2F). It is important to verify that it is effectively placed inferior to the level of the dorsum sellae, which would reduce drain displacement. As soon as the drain is correctly positioned the proximal part of the catheter need to be stitched to the skin to avoid inadvertent drain displacement. The bone flap can be replaced in case of a lax brain obtained at the end of the procedure also in accordance with Institutional Protocol (Figure 1F).

### Post-Operative Course

A postoperative CT scan is usually performed the day after surgery. We usually set the drainage in order to obtain around 5–10 cc of CSF per hour for 5–7 days after the surgical procedure. The amount of drainage can be adapted also according to the post-operative values of ICP. The amount of CSF drainage is gradually decreased and the cisternal drain is removed usually 7 days after surgery.

## Discussion

Cisternostomy represents a novel surgical strategy to treat severe TBI. There are now several publications that show encouraging results for cisternostomy over traditional methods of treatment for sTBI (4, 5, 7). Different surgical techniques have been described in the literature some including an extradural anterior clinoidectomy (and posterior clinoidectomy in some cases) (8) and other less aggressive ones (4, 5). However, there has an undue emphasis given to the complexity of the surgical approach which has generated resistance to this technique within the neurosurgical community, that is essentially undeserved (9). Therefore it is essential that this surgical technique is demystified. Based on the experience of our previously published surgical series we aimed to illustrate in details the surgical technique that is commonly used in our Institution. As presented in the cadaveric dissection and the illustrative intraoperative images, the first part of the surgery consists of a “trauma flap” in order to perform a fronto-temporo-parietal craniotomy/craniectomy that is extended till the middle fossa floor to ensure temporal lobe bony decompression (10). As illustrated in the figures and videos we routinely stop the extradural drilling at the level of the superior orbital fissure. In our experience performing an extradural anterior clinoidectomy is usually not necessary as also reported by other authors (7). The surgical technique of performing an extradural anterior clinoidectomy (EAC) demands specific technical skills, takes a certain amount of time that makes it poorly suitable in a context of urgent surgery and moreover can lead to well described complications in terms of vascular injury, thermic injury to the optic nerve, third nerve palsy and CSF leak (11). A proper basal craniotomy and a rapid extradural sphenoidal drilling till the superior orbital fissure can be rapidly accomplished with an acceptable extra-time of approximately 15–20 min when compared to a standard trauma flap. This slight increase in terms of amount of time has been found also by Chandra et al. (5). It is to be specified that this increased time includes the extradural sphenoidal drilling and the intradural phase of cisternal opening and positioning of the cisternal drain. An enough basal craniotomy, in general allows (12) access to the optico-carotid cistern, following the opening of which, the brain becomes much less tense and allows to complete the rest of the procedure. However, access to the cisterns is more challenging in case of a secondary surgical procedure (patients with refractory intracranial hypertension despite maximal medical therapy), and these cases may require increased surgical expertise and extradural work. The durotomy is also a critical step while dealing with surgeries for intracranial hypertension. Large durotomies will result in brain herniation with potential venous kinking and lacerations of the cortex on the bone edge and secondary brain injury leading to further brain swelling (13–15). To avoid these issues and to render possible an early access to the cisterns, it is mandatory to start with a very basal durotomy in proximity to the optico-carotid cistern. This small durotomy will avoid brain herniation and would leave the vast majority of the frontal lobe covered by the dura thus reducing iatrogenic contusions. If indicated, this small basal durotomy can be enlarged to access other lesions placed more posteriorly like hematomas or contusions. The opening of the Liliequist’s membrane allows a communication between the supratentorial and infratentorial basal cisterns. The anatomy of this membrane could be variable as nicely illustrated by Froelich et al. (16). Essentially three main configurations can be identified: one with a single membrane that starts from the arachnoid covering the dorsum sellae and ends between the infundibulum and mammillary bodies, one with a diencephalic leaf and a mesencephalic leaf that run in a separate course and the last one with a single membrane in the anterior part separating in two leaves in the posterior part. The dissection is then continued with the opening of the lamina terminalis that is part of our surgical technique and represents a modification of the initial technique as described by Cherian et al. (8). We believe that this surgical maneuver is important because it allows to drain both the ventricular and cisternal CSF thus resulting in a more efficient drainage, approximately a mean of 200 mL/day, as presented in our previous study. Moreover clinical experience from subarachnoid hemorrhage has showed that the fenestration of both lamina terminalis and Liliequist’s membrane is associated with a statistically significant reduction of shunt dependent hydrocephalus, probably because this maneuver relieve obstruction to CSF flow creating an alternative flow pathway (17). The final step of the procedure is represented by the positioning of a standard EVD catheter into the cisternal compartment under microscopic view. We introduce the drain into the optico-carotid cistern and then gently advance it towards the prepontine cistern. The decision to leave out or replace the craniotomy bone flap is open to interesting reflections. Although promising results have been published with cisternostomy as a stand-alone treatment (3, 5, 7), uncertainties are still present given the limited published clinical experience. A reasonable way to proceed would be not to replace the bone flap if the brain does not achieve a complete relaxation after cisternostomy or in case of borderline situations. This should also be done in accordance with Insitutional Protocols. In case of primary procedure, after the evacuation of a mass effect lesion (acute subdural hematoma) the decision whether to replace the bone flap or not is actually based on clinical and intraoperative parameters (18) and still subject to uncertainties that will be possibly clarified by the awaited results of the RESCUE-ASDH trial. The potential advantages of replacing the bone flap at the primary surgery avoids a future cranioplasty that have been reported to be associated with complications like infections, reoperations, extra-axial fluid collection, bone flap resorption and, scalp necrosis (19). In cases of surgery where cisternostomy is performed as a secondary procedure, the bone flap replacement is not always possible due to a more severe brain oedema in these patients. Results of all surgical therapies in this group are suboptimal. This is possibly a reflection of the severity of the trauma and a possible delay in treatment. Further clinical studies need to be done to critically evaluate the true benefits of maximal medical therapy and essentially determine the ideal timing of surgery.

## Conclusion

Cisternostomy represents a promising surgical technique in the setting of severe brain trauma. We present here a step-by-step description of the surgical anatomy along with cadaveric dissection to illustrate this technique. EAC and other complex surgical maneuvers are not usually required. The surgical procedure can be safely achieved in the majority of cases by surgeons who have had a basic neurosurgical training.

## Data Availability

The original contributions presented in the study are included in the article/Supplementary Material, further inquiries can be directed to the corresponding author/s.
