# Erratum: Basal cisternostomy for severe TBI: Surgical technique and cadaveric dissection

**DOI:** 10.3389/fsurg.2023.1183723

**Published:** 2023-03-22

**Authors:** Frontiers Production Office

**Affiliations:** Frontiers Media SA, Lausanne, Switzerland

**Keywords:** severe brain trauma, decompressive craniectomy (DC), skull base, cisternostomy, cadaveric dissection

An Erratum on Basal cisternostomy for severe TBI: Surgical technique and cadaveric dissection By Giammattei L, Starnoni D, Messerer M and Daniel RT. (2022) Front. Surg. 9:915818. doi: 10.3389/fsurg.2022.915818

An omission to the funding section of the original article was made in error. The following sentence has been added: “Open access funding provided by University of Lausanne”.

The original version of this article has been updated.

